# Where women go to deliver: understanding the changing landscape of childbirth in Africa and Asia

**DOI:** 10.1093/heapol/czx060

**Published:** 2017-05-25

**Authors:** Dominic Montagu, May Sudhinaraset, Nadia Diamond-Smith, Oona Campbell, Sabine Gabrysch, Lynn Freedman, Margaret E Kruk, France Donnay

**Affiliations:** 1 Department of Epidemiology and Biostatistics, Global Health Sciences, University of California, San Francisco; San Francisco, CA, USA,; 2 Community Health Sciences; University of California, Los Angeles; Los Angeles, CA, USA,; 3 London School of Hygiene and Tropical Medicine, London, UK,; 4 Heidelberg University, Heidelberg, Germany,; 5 Population and Family Health, Columbia University, New York, NY, USA; 6 Department of Global Health and Population, Harvard University, Cambridge, MA, USA; 7 School of Public Health and Tropical Medicine, Tulane University, New Orleans, Louisiana, USA

**Keywords:** Birth demographic shift, childbirth, developing countries, facility-based delivery, health care, home-delivery, location, maternal health, quality, safe delivery

## Abstract

Growing evidence from a number of countries in Asia and Africa documents a large shift towards facility deliveries in the past decade. These increases have not led to the improvements in health outcomes that were predicted by health policy researchers in the past. In light of this unexpected evidence, we have assessed data from multiple sources, including nationally representative data from 43 countries in Asia and Africa, to understand the size and range of changing delivery location in Asia and Africa. We have reviewed the policies, programs and financing experiences in multiple countries to understand the drivers of changing practices, and the consequences for maternal and neonatal health and the health systems serving women and newborns. And finally, we have considered what implications changes in delivery location will have for maternal and neonatal care strategies as we move forward into the next stage of global action. As a result of our analysis we make four major policy recommendations. (1) An expansion of investment in mid-level facilities for delivery services and a shift away from low-volume rural delivery facilities. (2) Assured access for rural women through funding for transport infrastructure, travel vouchers, targeted subsidies for services and residence support before and after delivery. (3) Increased specialization of maternity facilities and dedicated maternity wards within larger institutions. And (4) a renewed focus on quality improvements at all levels of delivery facilities, in both private and public settings.

Key Messages

- Most deliveries in developing countries are now happening in hospitals and clinics.
- This recent demographic shift has not led to the improvements in outcomes that were expected.
- The reason for this is continued poor quality care.
- The solution is a renewed focus on facility infrastructure, specialized services, access and both clinical and patient-centred support.

## Introduction

In 2015, 303 500 women died during childbirth and 2.7 million babies died within the first month of their life. The Millennium Development Goals (MDGs) built on widely shared calls for action to advocate for skilled birth attendance as a primary strategy to reduce such deaths, with rapid access to emergency services for complications. Health facility delivery helps ensure that women have access to skilled attendance, and as a result increasing the rate of facility delivery has been a central approach to improve maternal and neonatal health (Campbell and Graham 2006). Global demographic, economic and social changes in the past 15 years are changing delivery norms in many low- and middle-income countries. These changes are part of larger shifts in the context of health systems, include high rates of urbanization due to the influx of rural migrants to urban centres, modernization and development, changing gender norms, and changing social expectations. Their cumulative effect has led to over-burdened health systems in cities (Montgomery 2009; Matthews et al. 2010). Understanding if this new and growing burden also applies to facility deliveries, where, and across which population is important in program and policy planning given changing demographic trends.

Growing evidence from a number of countries has shown large shifts towards facility deliveries in the past decade. In many places, these increases have not, apparently, led to the improvements predicted. In light of this unexpected evidence, we have assessed nationally representative data from 43 countries to understand the size and range of changing delivery location in Asia and Africa. Many of the changes currently observed are driven by national policies as well as demographic factors. To put our findings into context, we have reviewed the policies, programs and financing experiences in multiple countries to understand the drivers of changing practices, and the consequences for maternal and neonatal health and the health systems serving women and newborns. And finally, we have considered what implications changes in delivery location will have for maternal and neonatal care strategies as countries around the world set out to address the Sustainable Development Goals and move forward towards Universal Health Coverage and into the next stage of global action.

## Methods

We used data from 43 Demographic and Health Surveys (DHS) collected between 2003 and 2013 to explore trends in facility deliveries, including all countries in Africa and Asia with at least two surveys during this period. We explored country and un-weighted regional changes in deliveries, and have broken down the results by wealth, location, place of delivery and more. Regions are defined by the United Nations standards for sub-regions. Significance testing was not done because of variations in time-between-surveys undertaken by DHS surveys in different countries. For a full list of countries and variables, please refer to a previously published paper in which the methods and variable selection are discussed in more detail (Diamond-Smith and Sudhinaraset 2015).

## Results

**Figure 1.**
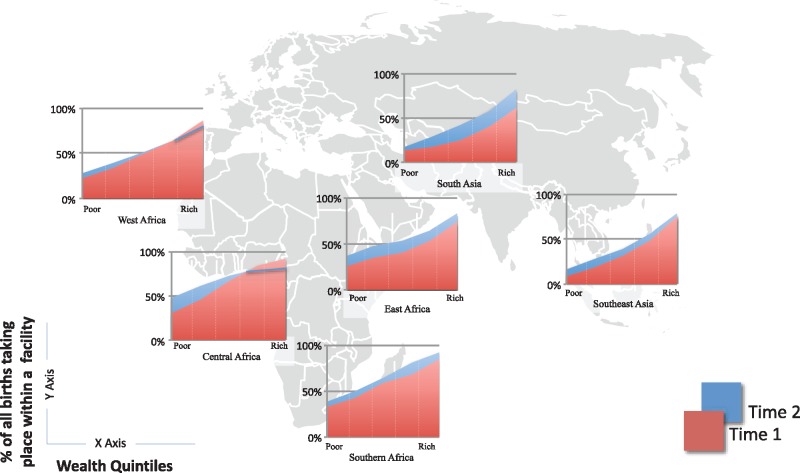
Trends in health facility deliveries across regions, by wealth quintiles (*Source*: Demographic and Health Surveys)

We found that in the past 15 years, health facility deliveries have increased in all regions, in almost all wealth groups (Figure 1). The only exception is among the richest populations in Central and West Africa where there has been a slight decline in the percent of deliveries facilities. Across regions, South Asia seems to lag behind other regions. This is driven primarily by India and Bangladesh, countries with historically high home birth rates. However, our analysis is based on the most recent public data, collected in India in 2005–6 before the Janani Suraksha Yojana (JSY) incentive program was implemented. Summary from the 2015–16 survey shows that facility deliveries have increased from 38.7% of all births in 2005–6 to 78.9% a decade later (“NFHS 4 Fact Sheet,” 2017). Once the data from this most recent survey are made public this change will be significant enough to make India, and all of South Asia, a leader in the shift to facility births.

There are significant poor–rich inequalities over time across all regions with the wealthier more likely to access facility care for deliveries. Rates of facility delivery are quite high among the wealthy, reaching over 80% in all regions except for South Asia at over 60%. The poorest had much lower rates ranging from 17% in Southeast Asia to a high of 48% in Central Africa.

**Figure 2.**
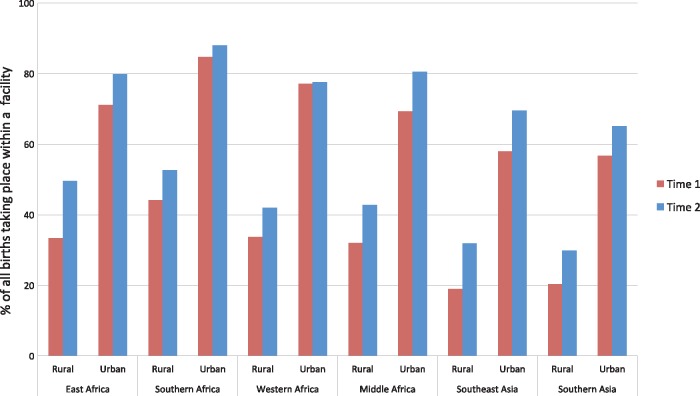
Trends in health facility deliveries across regions, by rural/urban (*Source*: Demographic and Health Surveys)

The shift towards facilities is also more than just a reflection of growing urbanization: the rate of health facility deliveries has increased in both urban and rural areas in all regions, save for stagnating rates in urban West Africa (Figure 2). In all parts of the world, women in urban areas are more likely to deliver in a facility than rural women, probably reflecting a combination of lack of availability of facilities, economic barriers to care, and social factors in rural areas.

**Figure 3.**
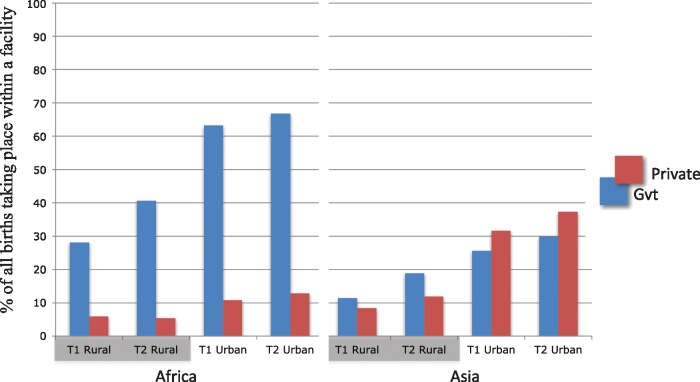
Public and private facility delivery trends across Africa and Asia, by urban/rural (*Source*: Demographic and Health Surveys)

Increases in facility deliveries have occurred in both public and private facilities, although there are regional differences (Figure 3). In Africa, the most dramatic increase occurred primarily in the public sector in rural areas, although the private sector has also increased everywhere except Southern Africa. In South and Southeast Asia, public and private sector facility deliveries have increased at similar rates. In these regions the private sector provides the majority of delivery care but is often not included in health system planning or data collection. This gap in policy and planning has significant implications as the private sector, heterogeneous and often unregulated, provides the majority of care for other maternal and paediatric health services as well.

**Figure 4.**
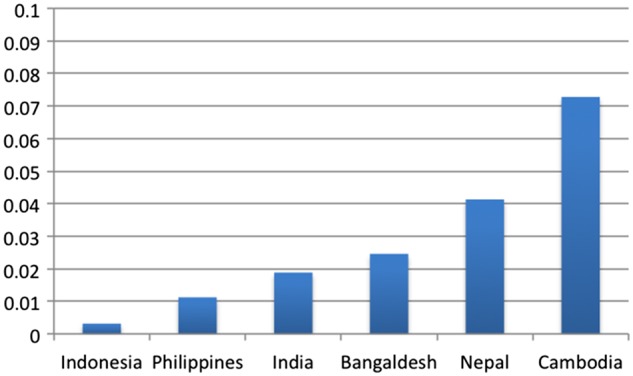
Annualized rate of change for each country in Asia sample (*Source*: Demographic and Health Surveys)

**Figure 5.**
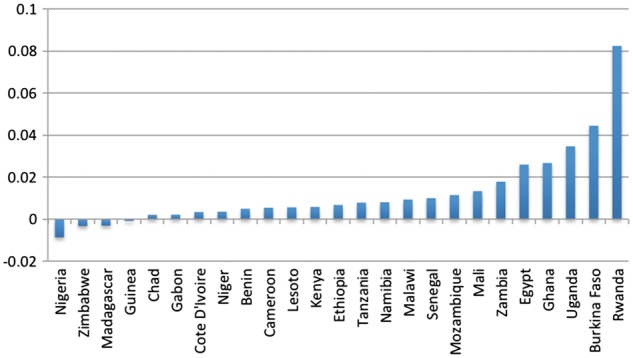
Annualized rate of change for each country in Africa sample (*Source*: Demographic and Health Surveys)

Within regions, rates of transition vary, but the trend towards increased delivery within facilities is widely shared. Annualizing the rate of change for each country in our sample (Figures 4 and 5) shows that while the proportion of health facility deliveries declined in a few countries, this has often happened in the context of larger economic or political turmoil (e.g. Zimbabwe). On the other end of the spectrum, some countries have experienced particularly rapid increases. In some instances (e.g. Cambodia) this high rate of shift was driven by a very low starting point.

## Policy context and discussion

This study finds that between 2003 and 2013, facility deliveries increased in all regions, in almost all wealth groups, particularly in rural areas, confirming the success of global strategies to increase skilled attendant deliveries (Campbell and Graham 2006). Our findings aggregate facility delivery trends across 10 years from 43 countries globally to assess broader trends in facility deliveries.

We also examined policy examples to contextualize the broader trends identified in the data. Major policy decisions may explain part of the upward trend in health facility deliveries, as evidenced by a few notable experiences in Asia and Africa. For instance, in Malawi and Rwanda, government regulations in the 2000 s greatly restricted access to assistance for home deliveries, effectively driving many women to deliver in facilities who might otherwise have preferred to remain at home (Cammack 2011; Bucagu et al. 2012; Chambers and Booth 2012; Banda 2013). In Rwanda, this policy was, from the start, linked to facility improvements in infrastructure and staff, lowered costs, better medicines, and information campaigns. In Malawi, many of those broader systems improvements came after the policy and are still being created. Although the policies on home-assistance restrictions in Malawi were ultimately rescinded, the effects remain: both countries went from <30% of deliveries in facilities at the turn of the millennium to, today, having >90% of all births delivered in clinics, hospitals and maternity homes. In other settings, for example Ghana, the introduction of free national insurance for pregnant women also led to increases in facility delivery (Dzakpasu et al. 2012).

Past studies have not addressed the mechanism by which global health efforts have shifted the number of women attending facility deliveries. Most of the work to understand factors associated with women delivering in a facility has emphasized individual demographic and household characteristics; however, accepted conceptual models suggest the importance of a wider range of determinants (Gabrysch and Campbell 2009; Moyer and Mustafa 2013; Diamond-Smith and Sudhinaraset 2015) including broader country and macro-level factors.

### Consequences for maternal and neonatal health and the health systems serving women and children

In India the introduction of a national conditional cash transfer program in 2005, JSY, had a transformative effect (Lim et al. 2010). A conditional cash transfer is a payment made to individuals who meet a predefined condition; in the case of JSY, giving birth in a facility. It is today the largest conditional cash transfer in the world. The scheme varies slightly from state to state but, broadly put, pays a cash incentive of between $15 and $30 to women who deliver in facilities, government-operated or private.

The effects of JSY in India have been both larger and more rapid than anyone dared imagine a decade ago: across all wealth quintiles, in urban and rural areas, the proportion of deliveries taking place in facilities has gone from below 20% of deliveries to above 70% in the past decade (Government of India 2012) and probably as high as 78.9% today (“NFHS 4 Fact Sheet,” 2017). Giving birth in a clinic or hospital has gone from a rarity to the norm, although inequalities, and large variations between states and regions, do still exist (Lim et al. 2010; Dongre and Kapur, 2013). But despite the improvement in access to care and improved access to referral systems which are implied by facility deliveries, maternal and neonatal mortality rates remain stubbornly high. This lack of effect is seen not only in India, but also in other countries where deliveries have shifted from home to facility just as rapidly (Randive et al. 2013). In wealthy OECD countries, a health facility delivery is highly protective for both mother and newborn (Koblinsky et al. 1999). Indeed, this well-documented protective effect is the reason why so much effort in Asia and Africa has focused on encouraging women to deliver in facilities rather than at home.

Why the same protective effect of facility delivery found in OECD countries is not apparent in low- and middle-income countries is not completely understood; however, some explanations are clear, and some theories seem likely. First, in many countries, the increase in facility deliveries has not been matched by an equivalent increase in the number of facilities, their staff, or their equipment. Clinics and health posts, often with pre-existing quality challenges, have been overwhelmed by increases in patient volume, and referral systems meant to assure that women with complicated deliveries move quickly from rural clinics to urban hospitals often do not work. A recent Lancet series highlighted the complexity of this issue, noting that both better infrastructure, and better, more nuanced, measurement of what drives behaviour are needed (Lieberman 2016).

While all governments recognize the need for multifaceted responses to high rates of maternal and neonatal mortality, lack of funding, lack of capacity and the quotidian challenges of policy changes mean that perfectly integrated responses are rare. More common is a step-wise introduction of some policies (i.e. cash incentives in India, restriction on traditional birth attendants in Malawi, user fee removals in many sub-Saharan countries) with a slower follow-up of quality improvements, transportation funds, communication and referral links, education outreach and the many other aspects of a holistic strategy. One consequence of this appears to have been, as noted above, that facility volumes increase first, and the health delivery system is left to catch up to the new reality.

Countries in which few women deliver in facilities may paradoxically have higher mortality rates among facility births than among home births. An explanation for this could be the effect of confounding in patient selection and bias in clinic reporting. When few women deliver in facilities, those who do are often those at risk or in need of urgent care, resulting in high rates of facility-reported mortality (Lohela et al. 2012). Also as noted by other researchers (Peters and Becker 1991), reporting of maternal deaths in clinics, even private clinics, is likely to always be better than at home, leading to perceived higher mortality rates in facilities. Together, these two characteristics of facilities—higher risk populations and higher reporting—may explain part of the high mortality associated with facility deliveries in low-facility-delivery countries.

At higher levels of facility use, when most children are born in a clinic or hospital, the confounding effects of patient self-selection should disappear, and may reverse: the remaining home deliveries being among populations too poor or distant from facilities to be able to choose an institutional delivery. In Europe, North America and the rest of the OECD, it seems that reporting differences between home and institutions are negligible, and that as nearly all births take place in facilities confounding from patient selection has no effect. When this is true, we see the protective effect of facilities clearly (Koblinsky et al. 1999).

In Asia and Africa, however, this is not uniformly the situation: in some countries where there are increasingly high levels of facility delivery, mortality remains high. Although home deliveries remain common in Sub-Saharan Africa, making up nearly half of all births, if facilities were protective, the increase in their use should lead to a decrease in mortality proportionate to their use. Assuming that the mortality rates are true and not the result of measurement, reporting or selection biases, we have to conclude that the reasons for the absence of improvement have to do with failures at the clinic and health system referral level.

### Implications for quality improvement

The implications of this have been noted elsewhere more than once: the quality of care offered in facilities to women giving birth and to their newborn children must improve (Clapham et al. 2004; Audo et al. 2005; Say et al. 2009; Pitchforth et al. 2010; Graham and Varghese 2012; Graham et al. 2013; Nesbitt et al. 2013;). There are many ways to do this, for example, through better preparedness for emergency obstetric cases (Paxton et al. 2005), better ongoing communication between staff members (Siassakos et al. 2009), integrated systems improvements (Merién et al. 2010), and financial incentives to providers, patients, and facility managers (Basinga et al. 2011) to name just a few. All have demonstrated positive results, and all are generally approved of by the global health community; however, the degree of active engagement has until now been underwhelming. Quality of care within facilities, which was important for the comparatively small numbers of women getting to facilities a decade or two ago is, we understand now, important for a much larger number of families as delivering in facilities is increasingly the norm rather than the exception for all women in all regions.

Improvements in facility staffing, infrastructure and services are at the heart of strategies for maternal and child health services in most countries. This is only a partial answer to quality failings, however (Koblinsky et al. 2016). Growing evidence shows that women with choices are able to judge poor quality, and increasingly often, react accordingly: studies across Africa have shown how women regularly bypass community clinics to reach first-level hospitals in search of quality services (Hansen et al. 2005; Kruk et al. 2009a, b, 2014). Across all parts of Asia and Africa, rapid improvements in roads and transport infrastructure mean that this trend to ‘bypass’ low-quality rural facilities in favour of larger, better options is likely to increase. Bypassing may be associated with the growth of private-facility-based deliveries in Asia and in rural Africa, particularly when private care is supported by newly added or expanded coverage under national health insurance programs (as in Ghana, Kenya and India), and by growing urban wealth, particularly in Asia. The widespread difficulty in assuring measurement of quality and adherence to quality regulations within private facilities contributes significantly to the variability of quality within private hospitals and within overall health systems where the private sector is large (Campbell et al. 2016; Montagu and Goodman 2016).

While small obstetric facilities in a number of OECD countries have proven able to assure high-quality outcomes (Nesbitt et al. 1990; Tracy et al. 2006), below some minimum number of deliveries per annum, staff skill levels and infrastructure readiness cannot be assured (Moster et al. 2001). This is becoming evident in low- and middle-income countries as well. Evidence from India and elsewhere (Banerjee et al. 2008) indicates the limitations on what quality is possible in rural low-volume facilities. Analysis of geospatial data from Zambia suggests that facilities in sparsely populated rural areas will be unable to obtain the number of deliveries thought necessary to maintain the skills of providers (Gabrysch et al. 2011). Compounding this problem, when most women with choices go elsewhere, the women with urgent need and no choice end up at the facilities with the least practiced providers, and consequently the greatest quality challenges.

These challenges are compounded by the complexity of what we summarize above as ‘quality’. Both the World Health Organization (WHO) and the United States’ Institute of Medicine (IOM) recognize six domains of quality, all with inherent value themselves, and all affecting women’s health-seeking behaviour, their access of medical care at an appropriate time, their understanding and compliance with medical advice, and their continuation of care for themselves and their children after giving birth. The domain of quality described above and determined by infrastructure, clinician skill, and facility volume are all components of Safety. But infrastructure, trained staff, and high volume do not, alone, lead to positive health outcomes (Souza et al. 2013). Assuring both outcomes and human rights requires attention to both safety and the other five domains of maternal health quality recognized by WHO: Effectiveness, Patient-Centeredness, Timely care, Efficiency and Equity (Tunçalp et al. 2015). Recognizing quality shortcomings is, not surprisingly given the complexity of the topic, much easier than addressing them.

### What next?

The accumulated evidence from our analysis of demographic surveys, recent field research and our first-hand knowledge of policy changes in a large number of settings leads us to the conclusion that the first tier of the three-tier-model (health post, health centre, hospital) of health system services and referrals is not the adequate place for deliveries and newborn care. Close-to-community care centres will continue to be important for many health services but not for deliveries. If safety and other aspects of quality cannot be maintained in such low-volume settings, if women do not want to access low-quality facilities, if transport opportunities increasingly exist, and if most women are already, or will soon be, giving birth in a facility, then it behooves governments and the international community to support models of care that respond appropriately to this new reality.

We propose a shifting of resources that will respond to current and forecasted changes. We make four major policy recommendations. (i) An expansion of investment in mid-tier facilities for delivery services and a shift away from low-volume rural delivery facilities. (ii) Assure access for rural women through funding for transport infrastructure, travel vouchers, targeted subsidies for services, and residence support before and after delivery. (iii) Specialization of maternity facilities and dedicated maternity wards within larger institutions. And (iv) a renewed focus on quality improvements at all levels of delivery facilities, in both private and public settings.

Many of these ideas have been presented before in one form or another. Some, such as dedicated maternity wards, are already enshrined in WHO standards, are the norm in OECD countries, and have been part of calls for global policy attention in previous articles in this journal (Feldman and Hurst 1987; Koblinsky et al. 2006). Others are agreed on, but in the process of refinement; for example the defining signal functions that must be performed regularly in order to assure a clinic’s readiness to offer obstetric care (Gabrysch et al. 2011). Nor is this the first time that changes in evidence have spurred a call for more attention and new strategies to address maternal and child health (Donnay 2000; Campbell and Graham, 2006; Koblinsky et al. 2016). We are encouraged by the knowledge that in the past, global policies *have* changed as a result of changing practices and more accurate understandings of need. The 1985 Nairobi Conference on Women led to a focus on and expansion of community health workers in many countries. The 1998 Health for All in the 21st Century initiative of WHO and the Millennium Development Goals provided a foundation of support to JSY and other national strategies aimed at increasing facility deliveries. Time and again the global community has proven itself attentive to new evidence on shifting care-seeking and treatment, and resulting in changes in the burden of disease for women and children.

Improvements to health occur within a system, and while our focus here is on delivery services, we recognize that the changes are contextual (Kruk et al. 2016). Health system improvements must respond to both the population served and where people go to access healthcare. The evidence shows clearly that maternal delivery norms have shifted in the past years, and that a matching shift in policies is needed to reap the health benefits which are now within reach. We need more research and more evidence, new policies should be piloted, their impact assessed, and adaptations appropriate for country context planned for. But most importantly, we need to acknowledge the changed environment and in response begin planning for changes in the health systems.

## References

[czx060-B1] Audo M, Ferguson A, Njoroge P. 2005 Quality of health care and its effects in the utilisation of maternal and child health services in Kenya. East African Medical Journal 82: 547–53. 16463747 10.4314/eamj.v82i11.9407

[czx060-B2] Banda EC. 2013. Stakeholders’ Perceptions Of The Changing Role Of Traditional Birth Attendants In The Rural Areas Of Central West Zone, Malawi: A Mixed Methods Study. Dissertation Unpublished.

[czx060-B3] Banerjee AV, Glennerster R, Duflo E. 2008 Putting a band-aid on a corpse: incentives for nurses in the Indian public health care system. Journal of European Economic Association 6: 487–500. 10.1162/JEEA.2008.6.2-3.487 PMC2826809 20182650

[czx060-B4] Basinga P, Gertler PJ, Binagwaho A 2011 Effect on maternal and child health services in Rwanda of payment to primary health-care providers for performance: an impact evaluation. The Lancet 377: 1421–8. 10.1016/S0140-6736(11)60177-3 21515164

[czx060-B5] Bucagu M, Kagubare JM, Basinga P 2012 Impact of health systems strengthening on coverage of maternal health services in Rwanda, 2000–2010: a systematic review. Reproductive Health Matters 20: 50–61. 10.1016/S0968-8080(12)39611-0 22789082

[czx060-B6] Cammack D. 2011 Local governance and public goods in Malawi. IDS Bulletin 42: 43–52.

[czx060-B7] Campbell OM, Graham WJ. 2006 Strategies for reducing maternal mortality: getting on with what works. The Lancet 368: 1284–99. [17027735] 10.1016/S0140-6736(06)69381-1 17027735

[czx060-B8] Campbell OMR, Calvert C, Testa A 2016 The scale, scope, coverage, and capability of childbirth care. The Lancet 388: 2193–208. 10.1016/S0140-6736(16)31528-8 27642023

[czx060-B9] Chambers V, Booth D. 2012. Delivering maternal health: why is Rwanda doing better than Malawi, Niger and Uganda? [WWW Document]. Overseas Dev. Inst. ODI. URL http://www.odi.org.uk/publications/6614-maternal-health-mortality-health-goverance-malawi-uganda-niger-rwanda, accessed 17 November 2013.

[czx060-B10] Clapham S, Basnet I, Pathak L, McCall M. 2004 The evolution of a quality of care approach for improving emergency obstetric care in rural hospitals in Nepal. International Journal of Gynecology Obstetrics 86: 86–97. 15207689 10.1016/j.ijgo.2004.03.013

[czx060-B11] Diamond-Smith N, Sudhinaraset M. 2015 Drivers of facility deliveries in Africa and Asia: regional analyses using the demographic and health surveys. Reproductive Health 12: 6. 25595063 10.1186/1742-4755-12-6 PMC4320522

[czx060-B12] Dongre AA, Kapur A. 2013. How is Janani Suraksha Yojana Performing in Backward Districts of India? (SSRN Scholarly Paper No. ID 2197248). Social Science Research Network, Rochester, NY.

[czx060-B13] Donnay F. 2000 Maternal survival in developing countries: what has been done, what can be achieved in the next decade. International Journal of Gynecology Obstetrics 70: 89–97. 10884537 10.1016/s0020-7292(00)00236-8

[czx060-B14] Dzakpasu S, Soremekun S, Manu A 2012 Impact of free delivery care on health facility delivery and insurance coverage in Ghana’s Brong Ahafo Region. PLoS One 7: e49430. 23173061 10.1371/journal.pone.0049430 PMC3500286

[czx060-B15] Feldman E, Hurst M. 1987 Outcomes and procedures in low risk birth: a comparison of hospital and birth center settings. Birth 14: 18. 3646887 10.1111/j.1523-536x.1987.tb01444.x

[czx060-B16] Gabrysch S, Campbell OM. 2009 Still too far to walk: literature review of the determinants of delivery service use. BMC Pregnancy Childbirth 9: 34. 19671156 10.1186/1471-2393-9-34 PMC2744662

[czx060-B17] Gabrysch S, Zanger P, Seneviratne HR, Mbewe R, Campbell OMR. 2011 Tracking progress towards safe motherhood: meeting the benchmark yet missing the goal? An appeal for better use of health-system output indicators with evidence from Zambia and Sri Lanka. Tropical Medicine International Health 16: 627–39. 21320245 10.1111/j.1365-3156.2011.02741.x

[czx060-B18] Government of India, 2012 Sample Registration System Office of the Registrar General & Census Commissioner, India. website: http://www.censusindia.gov.in/vital_statistics/SRS_Reports_2012.html. Accessed on May 15, 2017.

[czx060-B19] Graham WJ, McCaw-Binns A, Munjanja S. 2013 Translating coverage gains into health gains for all women and children: the quality care opportunity. PLoS Medicine 10: e1001368. 23335862 10.1371/journal.pmed.1001368 PMC3545868

[czx060-B20] Graham WJ, Varghese B. 2012 Quality, quality, quality: gaps in the continuum of care. The Lancet 379: e5–6 10.1016/S0140-6736(10)62267-2 21474173

[czx060-B21] Hansen K, McPake B, Nakamba P, Archard L. 2005 Preferences for hospital quality in Zambia: results from a discrete choice experiment. Health Economics 14: 687–701. 15619273 10.1002/hec.959

[czx060-B22] Koblinsky MA, Campbell O, Heichelheim J. 1999 Organizing delivery care: what works for safe motherhood? Bulletin of the World Health Organization 77: 399–406. 10361757 PMC2557673

[czx060-B23] Koblinsky M, Matthews Z, Hussein J 2006 Going to scale with professional skilled care. The Lancet 368: 1377–86. 10.1016/S0140-6736(06)69382-3 17046470

[czx060-B24] Koblinsky M, Moyer CA, Calvert C 2016 Quality maternity care for every woman, everywhere: a call to action. The Lancet 388: 2307–20. 10.1016/S0140-6736(16)31333-2 27642018

[czx060-B25] Kruk ME, Hermosilla S, Larson E, Mbaruku G. 2014 Bypassing primary care clinics for childbirth: a cross-sectional study in the Pwani region, United Republic of Tanzania. Bulletin of the World Health Organization 92: 246–53. 24700992 10.2471/BLT.13.126417 PMC3967574

[czx060-B26] Kruk ME, Kujawski S, Moyer CA 2016 Next generation maternal health: external shocks and health-system innovations. The Lancet 388: 2296–306. 10.1016/S0140-6736(16)31395-2 PMC5167371 27642020

[czx060-B27] Kruk ME, Mbaruku G, McCord CW 2009 Bypassing primary care facilities for childbirth: a population-based study in rural Tanzania. Health Policy Plan 24: 279–88. 19304785 10.1093/heapol/czp011

[czx060-B28] Kruk ME, Paczkowski M, Mbaruku G, de Pinho H, Galea S. 2009 Women’s preferences for place of delivery in rural Tanzania: a population-based discrete choice experiment. American Journal of Public Health 99: 1666–72. [CrossRef]Mismatch] 19608959 10.2105/AJPH.2008.146209 PMC2724466

[czx060-B29] Lim SS, Dandona L, Hoisington JA 2010 India’s Janani Suraksha Yojana, a conditional cash transfer programme to increase births in health facilities: an impact evaluation. The Lancet 375: 2009–23. 10.1016/S0140-6736(10)60744-1 20569841

[czx060-B30] Lohela TJ, Campbell OMR, Gabrysch S. 2012 Distance to care, facility delivery and early neonatal mortality in Malawi and Zambia. PLoS ONE 7: e52110. 23300599 10.1371/journal.pone.0052110 PMC3531405

[czx060-B31] Matthews Z, Channon A, Neal S 2010 Examining the “urban advantage” in maternal health care in developing countries. PLoS Medicine 7: e1000327. 20856899 10.1371/journal.pmed.1000327 PMC2939019

[czx060-B32] Merién A, van de Ven J, Mol B, Houterman S, Oei S. 2010 Multidisciplinary team training in a simulation setting for acute obstetric emergencies: a systematic review. Obstetrics Gynecology 115: 1021–31. 20410778 10.1097/AOG.0b013e3181d9f4cd

[czx060-B33] Montagu D, Goodman C. 2016 Prohibit, constrain, encourage, or purchase: how should we engage with the private health-care sector? The Lancet 388: 613–21. 10.1016/S0140-6736(16)30242-2 27358250

[czx060-B34] Montgomery M. 2009 Urban Poverty and Health in Developing Countries (No. 64(2)), Population Bulletin. Populations Reference Bureau. URL: http://www.igwg.org/pdf09/64.2urbanization.pdf. Accessed May 15, 2017

[czx060-B35] Moster D, Lie RT, Markestad T. 2001 Neonatal mortality rates in communities with small maternity units compared with those having larger maternity units. BJOG International Journal of Obstetrics Gynaecology 108: 904–9. 10.1111/j.1471-0528.2001.00207.x 11563458

[czx060-B36] Moyer CA, Mustafa A. 2013 Drivers and deterrents of facility delivery in sub-Saharan Africa: a systematic review. Reproductive Health 10: 40. 23962135 10.1186/1742-4755-10-40 PMC3751820

[czx060-B37] National Family Health Survey #4. Fact Sheet [WWW Document], 2017 URL http://rchiips.org/nfhs/pdf/NFHS4/India.pdf, accessed 16 March 17.

[czx060-B38] Nesbitt RC, Lohela TJ, Manu A 2013 Quality along the continuum: a health facility assessment of intrapartum and postnatal care in Ghana. PLoS ONE 8: e81089. 24312265 10.1371/journal.pone.0081089 PMC3842335

[czx060-B39] Nesbitt TS, Connell FA, Hart LG, Rosenblatt RA. 1990 Access to obstetric care in rural areas: effect on birth outcomes. American Journal of Public Health 80: 814–8. 2356904 10.2105/ajph.80.7.814 PMC1404977

[czx060-B40] Paxton A, Maine D, Freedman LP, Fry D, Lobis S. 2005 The evidence for emergency obstetric care. International Journal of Gynecology Obstetrics 88: 181–93. 15694106 10.1016/j.ijgo.2004.11.026

[czx060-B41] Peters DH, Becker S. 1991 Quality of care assessment of public and private outpatient clinics in Metro Cebu, the Philippines. International Journal of Health Planning and Management 6: 273–86.

[czx060-B42] Pitchforth E, Lilford R, Kebede Y 2010 Assessing and understanding quality of care in a labour ward: a pilot study combining clinical and social science perspectives in Gondar, Ethiopia. Social Science and Medicine 71: 1739–48. 20855142 10.1016/j.socscimed.2010.08.001

[czx060-B43] Randive B, Diwan V, De Costa A. 2013 India’s conditional cash transfer programme (the JSY) to Promote Institutional Birth: is there an association between institutional birth proportion and maternal mortality? PLoS ONE 8: e67452. 23826302 10.1371/journal.pone.0067452 PMC3694862

[czx060-B44] Say L, Souza JP, Pattinson RC. 2009 Maternal near miss—towards a standard tool for monitoring quality of maternal health care. Best Practice And Research Clinical Obstetrics and Gynaecology 23: 287–96. 19303368 10.1016/j.bpobgyn.2009.01.007

[czx060-B45] Siassakos D, Crofts J, Winter C, Weiner C, Draycott T. 2009 The active components of effective training in obstetric emergencies. BJOG International Journal of Obstetrics Gynaecology 116: 1028–32. 10.1111/j.1471-0528.2009.02178.x 19438497

[czx060-B46] Souza JP, Gülmezoglu AM, Vogel J 2013 Moving beyond essential interventions for reduction of maternal mortality (the WHO Multicountry Survey on Maternal and Newborn Health): a cross-sectional study. The Lancet 381: 1747–55. 10.1016/S0140-6736(13)60686-8 23683641

[czx060-B47] Tracy SK, Sullivan E, Dahlen H 2006 General obstetrics: does size matter? A population-based study of birth in lower volume maternity hospitals for low risk women. BJOG International Journal of Obstetrics Gynaecology 113: 86–96. 10.1111/j.1471-0528.2005.00794.x 16398776

[czx060-B48] Tunçalp ö, Were W, MacLennan C 2015 Quality of care for pregnant women and newborns-the WHO vision. BJOG International Journal of Obstetrics Gynaecology 122: 1045–9. 10.1111/1471-0528.13451 PMC5029576 25929823

